# A healthy dietary pattern with a low inflammatory potential reduces the risk of gestational diabetes mellitus

**DOI:** 10.1007/s00394-021-02749-z

**Published:** 2021-11-30

**Authors:** Lotta Pajunen, Liisa Korkalo, Ella Koivuniemi, Noora Houttu, Outi Pellonperä, Kati Mokkala, Nitin Shivappa, James R. Hébert, Tero Vahlberg, Kristiina Tertti, Kirsi Laitinen

**Affiliations:** 1 Institute of Biomedicine, Research Centre for Integrative Physiology and Pharmacology, University of Turku, 20520 Turku, Finland; 2 Department of Food and Nutrition, University of Helsinki, 00014 Helsinki, Finland; 3 Department of Obstetrics and Gynecology, University of Turku and Turku University Hospital, 20520 Turku, Finland; 4 Cancer Prevention and Control Program and Department of Epidemiology and Biostatistics, Arnold School of Public Health, University of South Carolina, Columbia, SC 29208 USA; 5 Department of Nutrition, Connecting Health Innovations LLC, Columbia, SC 29201 USA; 6 Institute of Clinical Medicine, Biostatistics, University of Turku, 20014 Turku, Finland

**Keywords:** Gestational diabetes mellitus, Dietary pattern, Dietary inflammatory index, Saturated fatty acid

## Abstract

**Purpose:**

An optimal diet for lowering the risk of gestational diabetes mellitus (GDM) is still to be defined, but may comprise of nutrient intakes, dietary patterns, diet quality, and eating frequency. This study was designed to investigate the contribution of diet in developing GDM in a comprehensive way.

**Methods:**

The dietary intake of overweight or obese women, a risk group for GDM (*n* = 351), was assessed using 3-day food diaries and diet quality questionnaires in early pregnancy. Eating frequency and nutrient intakes were calculated, and dietary patterns identified using principal component analysis. The inflammatory potential of the diet was determined by calculating the dietary inflammatory index (DII^®^) and energy-adjusted DII (E-DII™). GDM was diagnosed with an oral glucose tolerance test at 24–28 gestational weeks.

**Results:**

Higher adherence to ‘healthier dietary pattern’ characterized by consumptions of vegetables and rye bread associated with a reduced risk of GDM (adjusted OR 0.27, 95% CI 0.11–0.70). Higher E-DII score, indicating pro-inflammatory diet, was associated with a 27% higher risk of GDM (adjusted OR 1.27; 95% CI 1.08–1.49) for each E-DII point. In the evaluation of nutrient intakes, total fat, saturated fatty acids (SFAs), and trans fatty acids were higher and fiber lower in women developing GDM compared to women not developing GDM (all *p* < 0.05). Intakes of total fat, SFAs, and trans fatty acids were also significant predictors for GDM (all *p* < 0.05).

**Conclusions:**

The results emphasize the importance of an overall healthy diet and limitation of foods with SFAs, and other nutrients with a high inflammatory potential in reducing the risk of GDM.

**Trial registration:**

ClinicalTrials.gov Identifier: NCT01922791, August 14, 2013.

**Supplementary Information:**

The online version contains supplementary material available at 10.1007/s00394-021-02749-z.

## Introduction

Gestational diabetes mellitus (GDM) is a common condition in pregnancy, affecting almost 17% of all pregnancies worldwide [1]. GDM has both short- and long-term health effects on the mother and offspring; it increases the risk of pre-eclampsia and macrosomia [2–4], and predisposes mothers to type 2 diabetes [5] and children to obesity [6]. Along with obesity, lifestyle habits, primarily diet, have been implicated as key contributory factors in the onset of GDM [7, 8].

There is convincing evidence that diet has an important role in the onset of GDM. Intakes of specific nutrients, including saturated fatty acids (SFAs) and cholesterol, have been linked with an elevated risk of GDM [9–11], while fiber has been linked with a decreased risk of GDM [12, 13]. However, in some studies, these relationships have not been detected [14]. Dietary patterns, describing the dietary intake in a more comprehensive way, might clarify the relation of diet with the onset of GDM. Previous studies have indicated that a Western dietary pattern characterized with refined grains, high-fat dairy products, red meat, fast food, snacks, and sweets is associated with an elevated risk of GDM [15–18]. In contrast, a Mediterranean pattern and a healthy dietary pattern characterized with vegetables, rye bread, fruits and berries, and fish are associated with a decreased risk of GDM [18–20]. Again, some other investigators have not found any links between dietary patterns and the risk of GDM [21, 22]. The most recent suggestion has been that the inflammatory potential of the diet may be associated with various diseases [23] including GDM [24]. In addition to the composition of the diet, eating frequency, i.e., the number of meals per day and duration of overnight fasting time could contribute to the onset of GDM. Eating frequency has been shown to affect the glucose homeostasis of pregnant women [25], known to be an important factor in the pathophysiology of GDM [26]. The objective of this study was to investigate the extent to which diet in early pregnancy contributes to the risk of developing GDM in a high-risk group of women with overweight or obesity. We took a comprehensive approach and evaluated the relations between nutrient intakes, dietary patterns, overall diet quality, and eating frequency with the development of GDM. We also studied the inflammatory potential of the diet as inflammation may be a plausible mechanism for the onset of GDM.

## Methods

### Study design and subjects

We investigated, in a comprehensive way, how dietary intake in early pregnancy affects the onset of GDM in women participating in a clinical mother–infant dietary intervention trial. Each woman provided written informed consent before participation. The study has been described in detail previously [27]. Briefly, 439 overweight and obese (body mass index (BMI) ≥ 25 kg/m^2^) women, pregnancy at less than 18 gestational weeks were recruited in the study between 2013 and 2017. The study involved an intervention with fish oil, probiotics, both, or placebo. From our previous report [27], it was known that the intervention did not affect the onset of GDM, but it was nevertheless considered in the statistical analysis as a covariate. In this study, the relation of dietary intake in early pregnancy was evaluated in a comprehensive way in women developing GDM at later pregnancy compared to women not developing GDM. Background variables, use of dietary supplements, dietary intake from food diaries, and diet quality questionnaires were evaluated before or at the first study visit (mean 13.9, SD 2.1 gestational weeks). Women with completed food diary with or without completed diet quality questionnaire at early pregnancy, and an oral glucose tolerance test (OGTT) result at mid-pregnancy were included in this study, the final sample being 351 women (Online Resource 1).

### Diagnosis of GDM

GDM was diagnosed according to the Finnish Current Care Guidelines [28] and was based on the 2-h 75-g OGTT. The OGTT plasma glucose cut-off values were: fasting ≥ 5.3 mmol/l, 1-h ≥ 10.0 mmol/l and 2-h ≥ 8.6 mmol/l, which are in line with the American Diabetes Association 2007 Guidelines. The diagnosis was made if at least one of the values was high. OGTT was performed at 24–28 gestational weeks (median 25.9, IQR 25.0–27.0), but for high-risk women (GDM in a previous pregnancy, pre-pregnancy BMI > 35 kg/m^2^, glycosuria, type 2 diabetes in first degree relatives, oral corticosteroid medication, or polycystic ovary syndrome), the test was performed also at 12–14 gestational weeks. As our interest was the early pregnancy diet with reference to the onset of GDM, the women diagnosed with GDM in early pregnancy were excluded from the analyses.

### Food diaries

Three-day food diaries, including 2 weekdays and 1 weekend day, were kept during the week prior to the study visit. The women received oral and written instructions on how to fill in the diary. Participants were asked to record all foods and beverages consumed, and to estimate amounts using household measures, volumes, weight, or package labels. During the study visit, an illustrated portion picture booklet was used to check correctness of the diaries. Daily intakes of energy, energy-yielding nutrients, vitamins, and minerals from the diet were calculated using computerized software AivoDiet (version 2.0.2.3; Aivo, Turku, Finland) utilizing the Finnish Food Composition Database Fineli [29]. The nutrient intakes from dietary supplements were calculated using the manufacturer’s data sheets.

Eating frequencies of meals per day were calculated from the food diaries. Frequencies were determined for meals containing: (1) any food and/or beverages (total), (2) food with or without beverage, and (3) meals containing beverages only. However, zero-calorie drinks and foods (e.g., water, beverages with no energy, coffee and tea without sugar or milk, etc., and sugar-free pastilles) were omitted from investigation. The eating frequencies were compared to the recommended meal frequency, 4–6 meals/day, given by the Finnish Institute for Health and Welfare [30]. Overnight fasting time was calculated as an average of the two longest periods when the women were without food or energy-containing drink during the two nights. Only one available fasting period was also accepted to calculations.

### Dietary patterns

The list of the foods the women had eaten was extracted from the data and each food was classified into one of 88 food groups. The food grouping was based on the groups included in Finnish food composition database Fineli [29]. A mean consumption of each food group was calculated. Nutritionally similar groups were combined with each other, and 29 new food groups were formed (Online Resource 2). Of these, 24 groups were utilized in the dietary pattern analysis, since some of the groups were used by only a few women (e.g., substitutes for meat and dairy); the groups were difficult to combine with other groups or their consumption had not been marked in the diary accurately, e.g., water and spices (Online Resource 2).

Principal component analysis was used to reduce 24 food groups into a smaller number of components. The distributions of the food groups were not normal. However, because all the distributions were skewed in the same direction and the number of subjects was high, it was possible to conduct the principal component analysis. Received scree plot and Eigenvalues (> 1.5) were used in the selection of the components. The first two components were selected and rotated with Varimax rotation. Based on the loadings of different food group variables, these two components were translated into dietary patterns. Loadings > 0.10 and < -0.10 were reported. Each studied subject received component coefficient scores for both components. The subjects were divided into groups according to the quintiles (1–5) of both patterns, so that the highest quintile indicated the highest adherence to the pattern and vice versa.

### Overall diet quality

Validated index of diet quality (IDQ) questionnaire was utilized in the determination of the overall diet quality [31]. IDQ is an index depicting the dietary intake as a whole with reference to that recommended. The questionnaire includes 18 questions concerning the eating habits and consumption (including amount and frequency) of food groups: wholegrain bread, saturated/unsaturated fatty acids, dairy products, vegetables, fruits and berries, sugar containing drinks and sweets, and less than two skipped meals/week. After scoring each question, the diet was assessed to be of poor quality, if the index score was < 10, and of good quality if the score was ≥ 10 of the maximum 15 as defined in the article describing the validation of the index [31].

### Dietary inflammatory index (DII^®^)

The dietary inflammatory index, DII, is based on up to 45 food parameters which have been scored based on reported pro-inflammatory or anti-inflammatory effects on specific inflammatory markers (IL-1β, IL-4, IL-6, IL-10, TNF-α, and CRP) using 1943 peer-reviewed articles published through to December 2010. Details of the development and initial testing of the DII have been reported elsewhere [32, 33]. There have now been over 30 construct validation studies conducted all over the world using a variety of inflammatory biomarkers [34–36]. Briefly, the scoring algorithm uses a global reference database (food consumption from 11 populations globally) and food parameter-specific inflammatory effect scores to create an overall DII score for an individual. The DII scores an individual’s diet on a continuum from strongly anti-inflammatory (− 8.87) to strongly pro-inflammatory (+ 7.98).

When calculating DII scores of the participants in this study, dietary intake data were used to calculate an individual’s intake of food parameters which were then compared to the global reference database. A *Z*-score for each of the food parameters for each participant was calculated based on the global mean and standard deviation; this was achieved by subtracting the global mean from the amount reported and dividing this value by the standard deviation. The *Z*-scores were converted to a proportion (values 0–1) to minimise the effects of outliers (“right-skewing”). The standardised dietary intake data (proportion) were centred by doubling and subtracting 1. This value was then multiplied by the inflammatory effect score of each food parameter and summed to obtain an overall DII score for every participant in the study. A total of 28 food parameters were available from the food diaries in this study for computing the overall DII scores. These included energy, carbohydrate, protein, total fat, alcohol, fiber, cholesterol, SFA, monounsaturated fatty acids, polyunsaturated fatty acids, omega-3 fatty acids, omega-6 fatty acids, trans fatty acids, niacin, thiamine, riboflavin, vitamin B_12,_ vitamin B_6_, vitamin A, vitamin C, vitamin E, vitamin D, iron, magnesium, zinc, selenium, folic acid, and beta-carotene. In this study, we also computed the energy-adjusted DII (E-DII™) [37, 38]. This uses the same procedure as for the DII except that it employs an energy-adjusted global comparative database. In addition, because energy is in the denominator, only 27–1 food parameters were used in the calculation of the E-DII scores.

### Potential confounding factors

The information on mother’s background characteristics was gathered in detail before or during the first study visit. Mothers were asked about their education, lifestyle habits, previous pregnancies, and history of GDM. Additionally, their blood pressure was measured during the visit. BMI [weight (kg)/height (m)^2^] was calculated based on self-reported pre-pregnancy weight and height measured during the first study visit using a wall stadiometer.

A questionnaire was used to determine the physical activity [39]. The women were asked questions on their leisure-time physical activity during the previous week. The questionnaire covers the frequency, intensity, and duration of physical activity. A metabolic equivalent index for physical activity (MET-index, h/wk) was calculated and the MET-indexes were categorized into three classes based on the MET hours per week: light, moderate, and vigorous (< 5, 5–30 and 30 > MET h/week, respectively) [40].

### Statistical analysis

The data were checked visually from histograms and skewness was used to analyze the normality. Normally distributed continuous variables were summarized as means and standard deviations, and those that were not normally distributed were expressed as medians and interquartile ranges. Categorical variables were described as frequencies and percentages. In the comparisons, an independent-Samples *t* test was used if the data were normally distributed; otherwise, Mann–Whitney *U* test was utilized. Categorical variables were cross-tabulated, and Chi-Squared test or Fisher’s exact test was used to find differences. Correlations between DII, E-DII, and IDQ were investigated with Pearson correlation coefficient.

The association between nutritional factors, E-DII score and adherence to the dietary patterns, and the risk of GDM was examined using logistic regression models. Nutritional variables chosen in the models differed significantly between the women developing GDM and the women not developing GDM, and variables included in the same model did not correlate with each other (Spearman’s rho < 0.6). Absolute and energy-adjusted intakes of nutrients were included in the separate models; thus, overall nine models were constructed. In the models on dietary pattern, the first quintile was chosen to be a reference and p values were calculated across the quintiles (1–5). Models were adjusted with pre-pregnancy BMI, because it differed significantly between the women developing GDM and women not developing GDM, and the original trial intervention groups (probiotics and/or fish oil or placebo). The statistical analyses were conducted using SPSS, version 25 (IBM, New York, USA). *p* values < 0.05 were considered statistically significant in all of the tests.

## Results

### Subject characteristics

The characteristics of the women are shown in Table 1. Of the women, 23% (81/351) were diagnosed with GDM in late pregnancy (GDM +); thus, 77% did not develop GDM (GDM-). The women developing GDM had significantly higher pre-pregnancy BMI values and higher diastolic blood pressure, although within the normal reference range, compared to those not developing GDM. Neither physical activity (MET-index) nor categorized MET-index differed between the groups (Table 1).

**Table 1:** Early pregnancy clinical characteristics of the women developing or not developing gestational diabetes mellitus (GDM + /GDM−)

Characteristics	GDM +	GDM−	*p*^a^
	*n* = 81	*n* = 270	
Age (years)^b^	31.3 ± 4.5	30.3 ± 4.5	0.095
Primipara^c^	41 (51.2)	133 (49.3)	0.800
University or college education^c^	44 (55.0)	169 (65.5)	0.111
Blood pressure (mmHg) at early pregnancy study visit			
Systolic^d^	116 (111–123)	116 (110–123)	0.500
Diastolic^b^	78 ± 9.8	76 ± 7.8	0.026*
Pre-pregnancy BMI (kg/m^2^)^d^	29.9 (26.8–32.3)	28.4 (26.2–31.2)	0.041*
Overweight^c^	41 (50.6)	178 (65.9)	
Obese^c^	40 (49.4)	92 (34.1)	
Smoking before pregnancy^c^	11 (13.8)	60 (23.1)	0.084
Gestational weeks at early pregnancy study visit^b^	14.2 ± 2.0	13.8 ± 2.1	0.211
Previous GDM^c^	8 (9.9)	12 (4.4)	0.096
MET-index (h/wk)^d^	7.5 (3.0–12.0)	4.8 (3.0–12.0)	0.201
Categorized MET-index			0.171
Light physical activity^c^	34 (42.0)	143 (53.6)	
Moderate physical activity^c^	41 (50.6)	105 (39.3)	
High physical activity^c^	6 (7.4)	19 (7.1)	

*N* varies from 80 to 81 for GDM + and 258 to 270 for GDM−

*BMI* body mass index, *MET* metabolic equivalent index for physical activity

Overweight BMI < 30, Obese BMI ≥ 30

*Significant value (*p* < 0.05)

^a^Independent samples *t* test for normally distributed variables, Mann–Whitney *U* test for non-normally distributed variables and Chi-Squared test or Fisher’s exact test for categorical variables

Data are shown as ^b^mean ± SD, ^c^frequencies (percentages), ^d^median (IQR)

The characteristics between women included in the study and women excluded from the study are shown in Online Resource 3. The characteristics were similar, except that the excluded women had higher median pre-pregnancy BMI and more frequently GDM diagnosis in previous pregnancy compared to the women who were included in the study.

### Intakes of nutrients and development of GDM

The intakes of energy and energy-yielding nutrients are presented in Table 2. Whilst intake of energy did not differ between the women, the absolute intakes of total fat, SFAs, trans fatty acids, and the intake of SFAs as a proportion of energy intake were significantly higher in women developing GDM compared to those not developing GDM (Table 2). The intake of fiber expressed as nutrient density (grams per MJ) was significantly higher in women not developing GDM.

**Table 2:** Early pregnancy intakes of energy and energy-yielding nutrients in women developing or not developing gestational diabetes mellitus (GDM + /GDM−)

Nutrient	GDM +	GDM−	*p*^a^
	n = 81	n = 270	
Energy^b^			
MJ	8.50 ± 2.01	8.05 ± 1.92	0.072
Carbohydrates^b^			
E%	45.0 ± 6.58	46.0 ± 6.28	0.177
g	225 ± 62.3	218 ± 61.2	0.409
Protein			
E%	16.2 (14.3–18.6)^c^	16.7 (14.7–18.9)^c^	0.432
g	81.6 ± 20.7^b^	79.2 ± 21.3^b^	0.382
Fat			
Total fat			
E%	36.2 ± 6.43^b^	34.6 ± 6.54^b^	0.059
g	81.5 (67.9–93.3)^c^	74.9 (60.3–90.6)^c^	0.030*
SFAs			
E%	13.4 ± 2.79^b^	12.6 ± 3.05^b^	0.032*
g	29.4 (22.8–38.0)^c^	27.0 (21.1–32.7)^c^	0.014*
MUFAs			
E%	12.3 ± 2.96^b^	11.9 ± 2.70^b^	0.219
g	27.8 (21.4–32.7)^c^	25.0 (20.2–31.8)^c^	0.090
PUFAs			
E%	5.51 ± 1.61^b^	5.59 ± 1.56^b^	0.677
g	12.2 (9.28–15.0)^c^	11.9 (8.77–15.0)^c^	0.476
Trans fatty acids^c^			
E%	0.59 (0.50–0.71)	0.57 (0.47–0.68)	0.213
g	1.36 (1.01–1.75)	1.24 (0.92–1.53)	0.025*
Cholesterol^c^			
mg	225 (160–300)	223 (154–306)	0.892
Fiber^b^			
g/MJ	2.33 ± 0.61	2.57 ± 0.72	0.007*
g	19.6 ± 6.41	20.7 ± 7.64	0.238
Sucrose^b^			
E%	10.1 ± 3.86	10.5 ± 4.05	0.385
g	50.7 ± 23.2	50.2 ± 23.7	0.887

*E%* proportion of energy intake, *SFAs* saturated fatty acids, *MUFAs* monounsaturated fatty acids, *PUFAs* polyunsaturated fatty acids

*Significant value (*p* < 0.05)

^a^Independent samples *t* test for normally distributed variables and Mann–Whitney *U* test for non-normally distributed variables

Data are shown as ^b^ mean ± standard deviation or ^c^ median (interquartile range)

Intakes of vitamins and minerals are shown in Table 3. Significant differences were seen for the intakes of folate and vitamin C, expressed as nutrient densities (per MJ), being significantly lower in women developing GDM compared to women not developing GDM. Similarly, nutrient densities of magnesium, manganese, potassium, and phosphorus from the diet were lower among these women. When considering the intakes of nutrients from diet and supplements (total), the differences were seen in the intake of zinc being higher, with the intake of manganese (per MJ) being lower in women developing GDM. Overall, 94.3% (*n* = 331) of the women used at least one dietary supplement in early pregnancy. The most used supplement (71.2% of the women, *n* = 250) was multivitamin/multimineral for pregnancy (typically containing multitude of vitamins and minerals including vitamin D, folic acid, and iron, but not vitamin A). Vitamin D supplement was used by 23.6% of the women (*n* = 83), vitamin D + calcium by 6.6% (*n* = 24), calcium by 5.1% (*n* = 18), folic acid by 13.7% (*n* = 48), iron by 2.8% (*n* = 10), vitamin C by 4.3% (*n* = 15), and general multivitamin/multimineral (usually containing vitamin A) by 5.1% (n = 18), whilst none used iodine supplements. Other types of supplements were used only by few women (0.3–3.7%).

**Table 3:** Early pregnancy intakes (absolute and nutrient density) of vitamins and minerals from diet, and diet and supplements (total) of the women developing or not developing gestational diabetes mellitus (GDM + /GDM−)

Nutrient	GDM + *n* = 81		GDM− *n* = 270		*p*^a^	
	Diet	Total	Diet	Total	Diet	Total
Vitamin A						
μg	603 (456–751)	604 (461–751)	613 (486–831)	615 (487–865)	0.859	0.836
μg/MJ	72.2 (57.1–90.5)	73.4 (57.1–91.8)	78.9 (60.6–102)	79.0 (60.7–103)	0.139	0.134
Thiamine						
mg	1.19 (0.97–1.50)	4.45 (2.86–6.22)	1.18 (0.99–1.44)	4.11 (1.45–6.06)	0.706	0.052
mg/MJ	0.15 (0.13–0.18)	0.56 (0.32–0.75)	0.15 (0.13–0.17)	0.52 (0.19–0.73)	0.294	0.255
Niacin						
mg	29.8 (24.8–35.0)	47.4 (37.7–54.4)	29.2 (24.6–35.7)	44.4 (33.6–52.8)	0.719	0.077
mg/MJ	3.62 (3.05–4.23)	5.40 (4.61–6.63)	3.74 (3.25–4.25)	5.50 (4.31–6.68)	0.252	0.603
Folate						
μg	225 (192–292)	631 (540–715)	241 (195–296)	636 (484–742)	0.275	0.898
μg/MJ	27.5 (23.5–34.4)	76.0 (56.9–90.5)	30.4 (25.6–35.2)	78.0 (61.2–91.9)	0.019*	0.652
Pyridoxine						
mg	1.86 (1.52–2.31)	6.55 (3.84–7.29)	1.83 (1.45–2.25)	6.28 (2.27–7.10)	0.387	0.052
mg/MJ	0.23 (0.20–0.27)	0.74 (0.45–0.91)	0.23 (0.20–0.21)	0.73 (0.29–0.20)	0.902	0.457
Riboflavin						
mg	1.84 (1.30–2.20)	4.21 (2.92–6.87)	1.80 (1.41–2.21)	3.81 (2.12–6.58)	0.672	0.146
mg/MJ	0.21 (0.16–0.27)	0.49 (0.34–0.78)	0.22 (0.18–0.27)	0.49 (0.28–0.78)	0.050	0.486
Vitamin B12						
μg	4.44 (3.39–5.94)	7.13 (5.68–8.17)	4.33 (3.34–5.81)	6.66 (4.95–8.53)	0.831	0.272
μg/MJ	0.55 (0.38–0.72)	0.83 (0.69–1.04)	0.56 (0.44–0.72)	0.89 (0.64–1.09)	0.357	0.811
Vitamin C						
mg	116 (67.6–171)	213 (155–284)	131 (87.2–182)	213 (152–280)	0.150	0.715
mg/MJ	13.6 (8.68–21.2)	25.1 (18.9–36.1)	16.5 (11.2–22.2)	27.9 (18.7–35.0)	0.035*	0.606
Vitamin D						
μg	6.57 (4.85–10.5)	17.0 (13.3–23.4)	6.93 (4.84–10.1)	17.1 (13.5–23.8)	0.874	0.739
μg /MJ	0.86 (0.611–1.15)	2.10 (1.56–2.74)	0.90 (0.62–1.24)	2.24 (1.66–2.95)	0.375	0.603
Vitamin E						
mg	9.83 (7.92–12.2)	19.3 (14.4–23.6)	10.0 (7.76–12.3)	17.9 (12.5–22.6)	0.894	0.094
mg/MJ	1.22 (1.03–1.36)	2.33 (1.73–2.81)	1.22 (1.04–1.44)	2.32 (1.44–2.83)	0.244	0.610
Vitamin K						
μg	101 (74.9–128)	106 (76.8–131)	99.7 (73.5–135)	99.7 (73.5–135)	0.596	0.456
μg/MJ	12.2 (9.35–16.0)	12.4 (9.42–16.3)	12.1 (9.57–16.0)	12.2 (9.57–16.1)	0.881	0.960
Magnesium						
mg	316 (264–374)	427 (349–508)	308 (258–376)	420 (320–495)	0.985	0.186
mg/MJ	37.8 (32.2–42.4)	52.1 (41.3–63.0)	39.5 (34.4–44.8)	52.8 (42.7–62.1)	0.031*	0.823
Calcium						
g	1.07 (0.76–1.36)	1.15 (0.83–1.48)	1.02 (0.79–1.32)	1.09 (0.86–1.41)	0.735	0.970
g/MJ	0.13 (0.10–0.16)	0.13 (0.10–0.17)	0.13 (0.10–0.16)	0.14 (0.11–0.17)	0.162	0.129
Iron						
mg	10.6 (8.45–12.6)	26.4 (16.8–30.9)	10.5 (8.72–12.6)	23.5 (12.0–29.8)	0.997	0.069
mg/MJ	1.30 (1.10–1.44)	3.04 (1.87–3.61)	1.33 (1.16–1.49)	2.99 (1.47–3.66)	0.070	0.500
Chromium						
μg	19.6 (15.2–27.3)	21.3 (15.2–35.8)	19.2 (15.5–25.4)	21.6 (16.2–31.2)	0.648	0.875
μg/MJ	2.37 (1.97–3.01)	2.45 (2.01–3.91)	2.51 (2.05–3.00)	2.71 (2.13–3.57)	0.361	0.398
Copper						
mg	1.23 (0.93–1.48)	1.43 (1.05–2.00)	1.21 (0.97–1.44)	1.36 (1.04–1.92)	0.834	0.380
mg/MJ	0.15 (0.12–0.17)	0.16 (0.13–0.23)	0.15 (0.13–0.17)	0.16 (0.14–0.23)	0.158	0.920
Manganese						
mg	3.96 (3.18–5.16)	4.47 (3.28–5.96)	4.42 (3.40–5.77)	4.77 (3.69–6.29)	0.082	0.182
mg/MJ	0.49 (0.39–0.63)	0.52 (0.41–0.68)	0.57 (0.45–0.70)	0.59 (0.46–0.77)	0.003*	0.019*
Selenium						
μg	61.7 (51.9–76.3)	100 (73.5–119)	63.8 (52.1–76.7)	92.6 (65.3–113)	0.796	0.116
μg/MJ	7.89 (6.45–8.91)	11.7 (9.15–13.4)	8.00 (6.83–9.45)	11.2 (8.59–14.3)	0.167	0.836
Zinc						
mg	11.4 (9.03–13.1)	22.7 (17.6–26.4)	11.0 (8.97–12.5)	20.9 (12.1–26.0)	0.412	0.046*
mg/MJ	1.34 (1.15–1.49)	2.65 (1.93–3.30)	1.37 (1.20–1.52)	2.60 (1.47–3.24)	0.354	0.248
Salt						
g	6.70 (5.81–7.96)	–	6.47 (5.36–7.62)	–	0.189	–
g/MJ	0.84 (0.69–0.96)		0.82 (0.72–0.93)		0.750	
Phosphorus						
g	1.43 (1.18–1.64)	–	1.40 (1.16–1.64)	–	0.946	–
g/MJ	0.17 (0.14–0.20)		0.18 (0.15–0.20)		0.043*	
Sodium						
g	2.64 (2.28–3.09)	–	2.54 (2.10–3.00)	–	0.175	–
g/MJ	0.33 (0.27–0.37)		0.32 (0.28–0.37)		0.650	
Potassium						
g	3.43 (2.78–4.02)	–	3.45 (2.81–4.08)	–	0.937	–
g/MJ	0.41 (0.35–0.46)		0.43 (0.37–0.50)		0.037*	

Data are shown as medians (interquartile range)

^a^Mann–Whitney *U* test. *Significant value (*p* < 0.05)

We investigated the joint impact of different dietary factors in early pregnancy contributing to the development of GDM in late pregnancy in multivariable logistic regression models (Table 4 and Online Resource 4). In these models, after adjustments for pre-pregnancy BMI and intervention groups, higher intakes of total fat, SFAs, and trans fatty acids were significantly associated with an elevated GDM risk (Table 4).

**Table 4:** Associations between the joint effects of nutrient intakes and the risk of GDM

	OR^b^	(95% Cl)	*p*^a^
Model 1			
Total fat (g)^c^	1.12	(1.01–1.25)	0.027*
Zinc total (mg)^d^	1.04	(0.997–1.07)	0.072
Model 2			
SFA (g)^c^	1.42	(1.09–1.85)	0.009*
Zinc total (mg)^d^	1.04	(0.997–1.07)	0.068
Model 3			
Trans fatty acid (g)^d^	1.93	(1.17–3.20)	0.011*
Zinc total (mg)^d^	1.04	(0.997–1.07)	0.073

*Total* diet + supplements, *SFAs* saturated fatty acids, *OR* odds ratio, *CI* confidence interval

*Significant value (*p* < 0.05)

^a^Multivariable logistic regression model. Nutrients chosen in the models were in the same unit (absolute intake), and did not correlate with each other (Spearman rho < 0.60), and intakes differed significantly between the women developing GDM and women not developing GDM

^b^Adjusted for pre-pregnancy BMI and original trial intervention groups; OR for ^c^ten-unit or ^d^one-unit increase in continuous factors

### Food groups, dietary patterns, and development of GDM

**Fig. 1:**
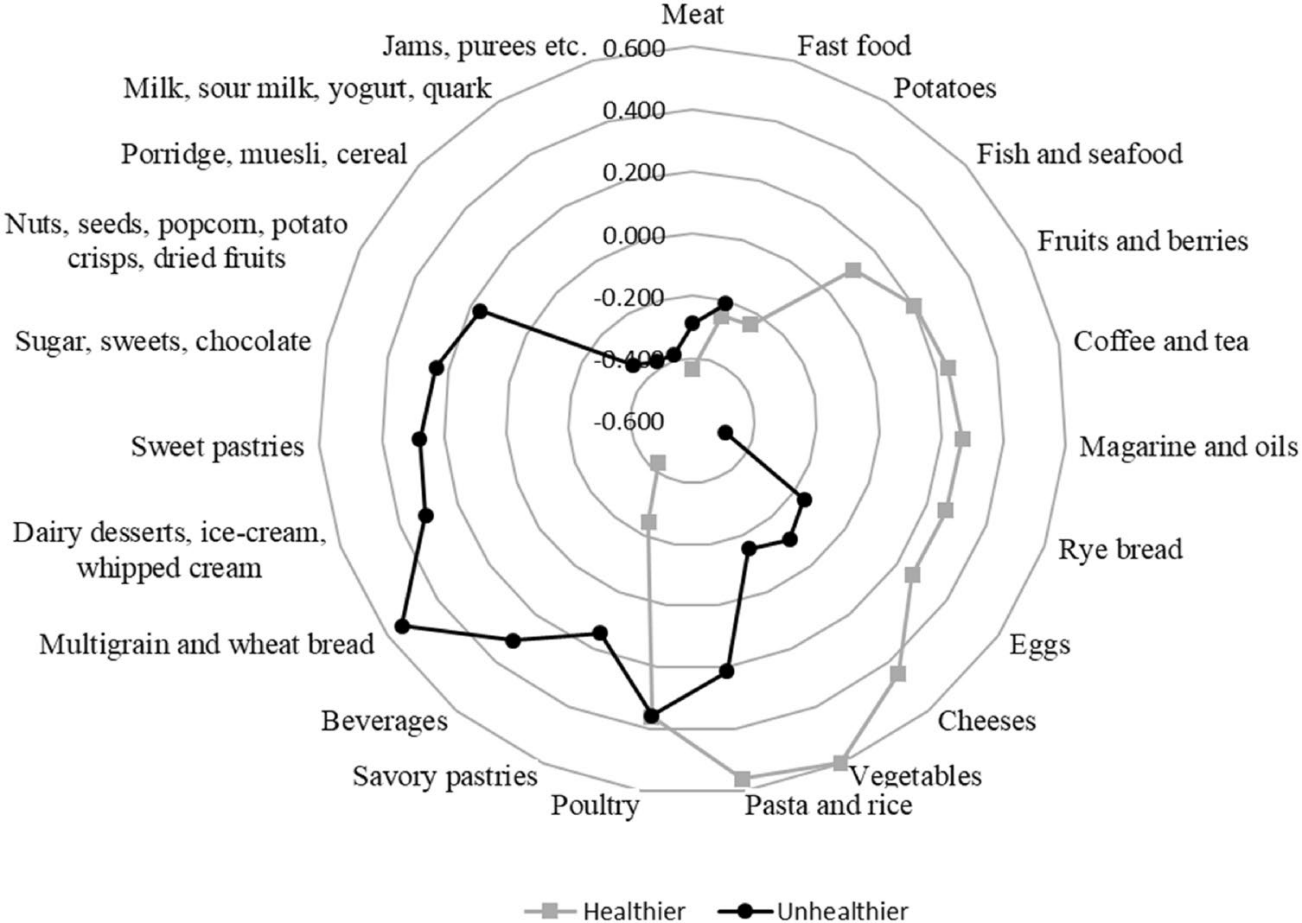
Dietary patterns derived with principal component analysis from food groups that were obtained from 3-day food diaries. Factor loadings (> 0.10 and < 0.10) of food groups of a healthier and an unhealthier dietary pattern

Two dietary patterns were identified from the consumed food groups and named as a healthier pattern and an unhealthier pattern. The components explained 8.0% and 7.1% of the total variance in the data. The healthier pattern was characterized by a high consumption of vegetables, fruits and berries, rye bread, pasta and rice, fish and seafood, coffee and tea, poultry, margarine and oils, cheeses, and eggs. The unhealthier pattern was characterized by a higher consumption of multigrain and wheat bread, dairy desserts (e.g., ice-cream), sweet pastries, savory pastries, sugar, sweets and chocolate, nuts, seeds, popcorn, potato crisps and dried fruits, beverages, pasta and rice, and poultry (Fig. 1). Loadings of the food groups are shown in Fig. 1.

In logistic regression model (Table 5), higher adherence (quintile 4) to the healthier dietary pattern was discovered to be related with a lower risk of developing GDM after adjustment with pre-pregnancy BMI and intervention groups. In contrast, the unhealthier pattern was not associated with an outcome.

**Table 5:** Association between adherence to the healthier or unhealthier dietary pattern and the risk of developing GDM

	GDM + *n* (%)	GDM− *n* (%)	Adjusted ^b^ OR (95% CI)	*p*^a^
Healthier diet				
1 (Lowest)	21 (25.9)	49 (18.1)	1 (Reference)	
2	15 (18.5)	55 (20.4)	0.65 (0.30–1.41)	0.277
3	21 (25.9)	50 (18.5)	0.94 (0.45–1.96)	0.872
4	7 (8.6)	63 (23.3)	0.27 (0.11–0.70)	0.007*
5 (Highest)	17 (21.0)	53 (19.6)	0.82 (0.38–1.74)	0.602
Unhealthier diet				
1 (Lowest)	18 (22.2)	52 (19.3)	1 (Reference)	
2	17 (21.0)	53 (19.6)	0.88 (0.40–1.91)	0.746
3	15 (18.5)	56 (20.7)	0.74 (0.33–1.64)	0.458
4	11 (13.6)	59 (21.9)	0.49 (0.21–1.15)	0.101
5 (Highest)	20 (24.7)	50 (18.5)	1.04 (0.48–2.22)	0.928

*Lowest adherence* 1st quintile, *highest adherence* 5th quintile, *OR* odds ratio, *CI* confidence interval

*Significant value (*p* < 0.05)

^a^Multivariable logistic regression models

^b^Adjusted for pre-pregnancy BMI and original trial intervention groups

### Dietary inflammatory potential and development of GDM

The energy-adjusted DII score, but not unadjusted DII, was significantly higher in women developing GDM compared to those not developing GDM, indicating that the inflammatory potential of the diet in women developing GDM was higher (Table 6). A higher E-DII was associated with a higher risk of developing GDM also after adjustments for pre-pregnancy BMI and intervention groups in logistic regression model.

**Table 6:** Dietary inflammatory index (DII) and energy-adjusted DII (E-DII) scores of women developing or not developing gestational diabetes mellitus (GDM + /GDM−)

	GDM + *n* = 81	GDM− *n* = 270	*p*^a^
DII score (mean ± SD)	− 0.31 ± 1.66	− 0.60 ± 1.77	0.191
E-DII score (mean ± SD)	− 0.70 ± 1.59	− 1.33 ± 1.56	0.002*

E-DII (continuous)	**OR**	**95% CI**	***p***^**a**^
Adjusted OR^b^	1.27	(1.08–1.49)	0.003*

The association between the E-DII score and the risk of developing GDM

*CI* confidence interval, *DII* dietary inflammatory index, *E-DII* energy-adjusted dietary inflammatory index, *OR* odds ratio

*Significant value (*p* < 0.05)

^a^Independent samples t test for group comparisons and multivariable logistic regression model for association between E-DII score and the risk of GDM

^b^Adjusted for pre-pregnancy BMI and original trial intervention groups; OR for one-unit increase in continuous factors

### Dietary quality and development of GDM

Mean dietary quality scores were 9.4 (SD 2.1) and 9.6 (SD 2.1) in women developing GDM and those not developing GDM, respectively, and no difference was seen between the groups (*p* = 0.51). An overall dietary quality was good in 42.5% of the women developing GMD and in 50.2% of the women not developing GDM with no difference (*p* = 0.25). We also investigated if dietary quality would be associated with the inflammatory potential of diet, and found that the IDQ correlated significantly both with DII (*r* = − 0.39, *p* ≤ 0.001) and E-DII (*r* = − 0.41, *p* ≤ 0.001).

### Eating frequency, overnight fasting time, and development of GDM

On average, the women developing GDM consumed 5.4 (SD 1.2) and those not developing GDM consumed 5.5 (SD 1.2) meals containing food with or without beverages, per day (*p* = 0.16), the majority having a recommended (4–6 meals/day) eating frequency (GDM + 69.1% and GDM − 72.7%, *p* = 0.79). No differences between women developing GDM (mean 5.7, SD 1.3) and those not developing GDM (mean 5.8, SD 1.3) were seen in the consumption frequency of meals consisting of any food and/or beverage (*p* = 0.37). The majority of the women did not have meals consisting only of an energy-containing beverage, and no difference was seen between the groups (*p* = 0.88). The mean overnight fasting time was the same in both groups (11.4 h, SD 1.7) and there was no significant difference (*p* = 0.86).

## Discussion

This prospective clinical study investigated the contribution of diet in developing GDM in a comprehensive way. Our study revealed that a higher adherence to the heathier dietary pattern was associated with a lower risk of GDM, and a high inflammatory potential of the diet (measured by E-DII) was associated with an increased risk of GDM. In the evaluation of the intakes of nutrients, higher intakes of total fat, SFAs, and trans fatty acids were significant predictors for an elevated GDM risk. In contrast to expectations, we found no association between eating frequency or overnight fasting time and the onset of GDM.

Our study, together with previous reports, indicates that a healthy diet reduces the risk of GDM. We found that a higher adherence to the healthier dietary pattern characterized, for example, by rye bread, vegetables, fruits, and berries was associated with a decreased risk of developing GDM. In a previous study [20] in 168 normal weight, overweight, and obese women, a prudent dietary pattern, including vegetables, seafood, and pasta, was associated with a decreased risk of GDM. Additionally, the relationship was confirmed when only women with overweight or obesity were included in the analysis. Further support is evident from a prospective cohort study [19], in which four different dietary patterns during pregnancy (traditional, sweet food, fried food-beans, and whole grain-seafood) were identified from data collected from 1014 women. Of these, a traditional pattern, resembling the healthier pattern of our study, characterized by vegetables, fruits, and rice was associated with a decreased risk of GDM. Additionally, in another large (*n* = 3853) study [18], four pre-pregnancy dietary patterns (Meats, snacks and sweets, Mediterranean-style, Cooked vegetables, Fruit, and low-fat dairy) were identified. They found that a Mediterranean pattern, including increased consumption of vegetables, nuts, and rye bread, was associated with a decreased risk of GDM. These findings altogether support the idea that an overall healthy diet is of importance in decreasing the risk of developing GDM, although, compared to our study (36 E%), fat intake was higher (40 E%), e.g., in the Mediterranean diet pattern [18]. Interestingly, in our study, it was found that higher adherence (4th quintile), but not the highest adherence (5th quintile) to the healthier diet, was associated with a lower GDM risk. The reason for the lack of association for the highest quintile remains unknown, calling for further investigations. Also, we did not detect any difference in an overall dietary quality, as measured by IDQ, between the women developing GDM and those not developing GDM. Although IDQ is a validated tool, it measures an overall diet quality by a short stand-alone questionnaire in reference to diet recommendations, whilst dietary patterns were determined based on the intakes of food groups calculated from food diaries, thus likely providing more detailed description of the dietary intake.

Considering the mechanisms behind the pathophysiology of GDM, it seems that low-grade inflammation as a mediator for insulin resistance [41] could contribute to the onset of GDM, particularly in overweight and obese women, as obesity promotes inflammation in the body and is a known risk factor for GDM [7]. Diet may act as a major contributor, because various foods and nutrients are associated with elevated levels of inflammatory markers [42]. The findings from our study support this concept as a higher E-DII score, i.e., a more inflammatory diet, was found to be associated with an elevated GDM risk. Our finding is in accordance with two prior studies, indicating that a higher DII score is associated with an increased risk of GDM [24] particularly in women with overweight or obesity [43]. The lower inflammatory potential of foods could partially explain the observed relation between the healthier dietary pattern and a reduced risk of GDM in our study [42]. Food groups, such as rye bread, vegetables, fruits and berries, fish, margarine high in unsaturated fatty acids, and vegetable oils, had high loadings in the healthier pattern. These food groups are rich in vitamins, minerals, fiber, and/or unsaturated fatty acids, and thus have a low inflammatory potential. Indeed, intakes of vitamin C, folic acid, and magnesium, nutrients with lower inflammatory potential [44–46], were higher in women not developing GDM compared to women developing GDM, although they were not associated with a risk of GDM in the logistic regression models. Interestingly, E-DII and DII correlated negatively with IDQ, again indicating that an overall healthier diet also is less inflammatory. Another mechanism how nutrients could affect the development of GDM is their effects on glucose and insulin metabolism as these are important factors in the pathophysiology of GDM [26]. For example, fiber intake has been shown to have beneficial effects on these, as well as body weight and satiety [47]. Although, in our study, only a small difference was seen in the intake of fiber between the women developing and not developing GDM, the previous studies have indicated that higher fiber intake is associated with a lower risk of GDM [12, 13]. Furthermore, the intake of salt [48] may unfavorably affect glucose and insulin metabolism, although no difference in salt intake was detected in our study.

In a more detailed examination of diet, we demonstrated that a higher intake of dietary fats is associated with an elevated GDM risk. More closely, the intakes of SFAs, and trans fatty acids were significantly higher in women developing GDM compared to women not developing GDM. Dietary fats could be one explanatory factor behind the relationship between inflammation and the onset of GDM. Total fat, SFAs, and trans fatty acids have a high inflammatory potential [32]; thus, they are able to increase the presence of chronic, systemic inflammation [42]. Our findings are in line with previous evidence, indicating that the quality of fat is an important factor in the development of GDM [9, 10]. Previous studies have linked a high intake of SFAs in early pregnancy with an elevated risk of developing GDM [9, 10]. Additionally, one study [49] found that women diagnosed with GDM had higher intakes of SFAs, and trans fatty acids as a percent of energy than women without GDM, and that total fat intake as a percent of energy was associated with an elevated GDM risk. More detailed breakdown of the consumed foods, e.g., based on source of protein, i.e., animal or vegetable protein could yield further insight into the onset of GDM, as indicated by a study in which higher intake of animal protein was associated with an increased GDM risk, while intake from vegetable source was associated with a decreased risk [50]. In another study, intake of total protein and animal protein increased the risk of GDM but no relation was found with intake of vegetable protein [51]. Also, evidence from a meta-analysis indicates that higher levels of circulating branched-chain amino acids may be associated with an increased risk of GDM [52].

We found no differences in eating frequency or overnight fasting time between the women developing GDM and those not developing GDM. No previous studies have investigated the association between eating frequency and the development of GDM. We hypothesized that eating frequency could contribute to the risk of GDM through its effect on glucose metabolism. Human bodies comply with a 24-h cycle, i.e., circadian clock regulated by light–dark cycle. Various physiological events such as insulin and glucose metabolism follow daily light–dark cycle. It has been shown that eating according to circadian clock, i.e., food intake during light phase and fasting during dark phase, has beneficial effects on the glucose regulation. Also, proper fasting intervals between the meals may enhance glucose homeostasis. [53] Hence, an adequate night-fasting period in addition to lower numbers of meals per day could decrease the risk of developing GDM through their effects on glucose metabolism. This relationship has previously been postulated by Loy and colleagues in pregnant women [25]. They found that each additional meal was associated with an increased 2-h post-prandial glucose level, while an hourly increase in overnight fasting period was associated with lower fasting glucose in pregnant women. It is of note that population in these two studies differed, which may partly explain the differing study results. It does seem that the relationship between eating frequency and development of GDM requires further investigation.

This study has various strengths; we evaluated a relatively large number of pregnant women in a prospective clinical study setting. This also allowed a detailed collection on women’s background and lifestyle habits, which were taken into account in “Statistical analysis”. Moreover, because the dietary assessment was conducted in early pregnancy, the temporal relationship between dietary intake and the risk of developing GDM was readily observable. We also used multiple tools to assess dietary intake; total energy intake, energy-yielding nutrients, nutrients from dietary supplements, dietary patterns, index of diet quality, and the inflammatory potential of the diet. This comprehensive evaluation of the women’s diets made it possible to find putative connective factors between the early pregnancy diet and the development of GDM later in her pregnancy.

Despite its strengths, this study has some limitations. First, because all subjects were either obese or overweight, the results can be generalized only for that population. Nonetheless, we took into account pre-pregnancy BMI by adjusting in the analyses; thus, the results may be potentially generalized to an overall population of pregnant women in Finland. Nevertheless, as obese women are more prone to develop GDM compared to normal-weight women and obesity is becoming more of a global problem, it is important to increase our knowledge in this population. Second, one possible source of error can be traced from underreporting of dietary intake. It is known that especially women with overweight or obesity tend to underreport their dietary intake [54]. Usually, underreporting focuses on high-caloric foods, such as fat and sugary foods, and thus could affect estimation of total energy and nutrient intakes [54]. Also, evaluation of eating behavior domains [55] could bring insight into the diet GDM associations. Third, the excluded women had higher median pre-pregnancy BMI and more frequently GDM diagnosis in previous pregnancy compared to women included in the study. Thus, this might have influenced the results as the included women were generally healthier than the excluded women. A fourth limitation arises from the analysis for defining dietary patterns. Two components explained about 15% of the total variance in the data, which is a relatively low value. However, similar percentages are also described in other studies depending on the number of components selected. In one previous study, one component explained 8.2% of the total variance [20], while in another study, four components explained 45.6% of the total variance [56]. In our study, the 4th quintile, but not the 5th quintile, was associated with a lower risk of GDM. The finding could relate to the study technical issues, e.g., study power to detect differences between the quintiles. However, our finding is supportive to the findings from other studies, which indicate that an overall healthier diet, and also lower intake of nutrients that have a capacity to promote inflammation, such as SFA, could decrease the risk of GDM. It is noteworthy that, in addition to diet, other factors such as obesity, age, and previous GDM are involved in the development of GDM. Additionally, because healthy lifestyle markers typically cumulate, it is possible that the women whose diet is healthy also exercise and sleep more, which might contribute to the association between diet and development of GDM. Finally, in addition to inspecting dietary fat and carbohydrate quality by separating intakes of SFA, MUFA, PUFA, trans fatty acids, cholesterol, and fiber, and sucrose, evaluating food sources of these nutrients could have provided deeper insight into the onset of GDM. Overall, our findings are in line with those in the literature; a healthy diet with anti-inflammatory effects decreased the risk of GDM, while a higher intake of nutrients with a pro-inflammatory potential, total fat, SFAs, and trans fatty acids increased the risk of GDM.

Taken together, the results suggest that pregnant women with overweight or obesity consuming an overall healthy diet and one with a low inflammatory potential experience a lower risk of developing GDM compared to their counterparts who consume a more pro-inflammatory, less healthy diet. It was also demonstrated that avoiding an excess intake of dietary fats, especially SFAs, and other nutrients promoting inflammation in the body, might be associated with a decreased risk of developing GDM. It is likely that considering these aspects in the dietary counselling would benefit these women.

## Supplementary Information

Below is the link to the electronic supplementary material.

Supplementary file1 (DOCX 30 KB)

Supplementary file2 (DOCX 17 KB)

Supplementary file3 (DOCX 21 KB)

Supplementary file4 (DOCX 19 KB)

## Data Availability

Data are not available.

## Abbreviations

BMI: Body mass index
DII: Dietary inflammatory index
E-DII: Energy-adjusted dietary inflammatory index
GDM: Gestational diabetes mellitus
IDQ: Index of diet quality
MET: Metabolic equivalent index for physical activity
OGTT: Oral glucose tolerance test
SFA: Saturated fatty acid

## References

[1] International Diabetes Federation. Gestational diabetes. https://idf.org/our-activities/care-prevention/gdm.html. Accessed 3 Mar 2021

[2] Köck K, Köck F, Klein K, Bancher-Todesca D, Helmer H (2010). Diabetes mellitus and the risk of preterm birth with regard to the risk of spontaneous preterm birth. J Matern Fetal Neonatal Med.

[3] Mohammadbeigi A, Farhadifar F, Soufi Zadeh N, Mohammadsalehi N, Rezaiee M, Aghaei M (2013). Fetal macrosomia: risk factors, maternal, and perinatal outcome. Ann Med Health Sci Res.

[4] Yogev N, Chen N, Hod N, Coustan N, Oats N, McIntyre N, Metzger N, Lowe N, Dyer N, Dooley N, Trimble N, McCance N, Hadden N, Persson N, Rogers N (2010). Hyperglycemia and Adverse Pregnancy Outcome (HAPO) study: preeclampsia. Am J Obstet Gynecol.

[5] Herath H, Herath R, Wickremasinghe R (2017). Gestational diabetes mellitus and risk of type 2 diabetes 10 years after the index pregnancy in Sri Lankan women-A community based retrospective cohort study. PLoS ONE.

[6] Reece EA (2010). The fetal and maternal consequences of gestational diabetes mellitus. J Matern Fetal Neonatal Med.

[7] Chu SY, Callaghan WM, Kim SY, Schmid CH, Lau J, England LJ, Dietz PM (2007). Maternal obesity and risk of gestational diabetes mellitus. Diabetes Care.

[8] Mijatovic-Vukas J, Capling L, Cheng S, Stamatakis E, Louie J, Cheung NW, Markovic T, Ross G, Senior A, Brand-Miller JC, Flood VM (2018). Associations of diet and physical activity with risk for gestational diabetes mellitus: a systematic review and meta-analysis. Nutrients.

[9] Mizgier M, Jarzabek-Bielecka G, Mruczyk K (2019). Maternal diet and gestational diabetes mellitus development. J Matern Fetal Neonatal Med.

[10] Meinilä J, Koivusalo SB, Valkama A, Rönö K, Erkkola M, Kautiainen H, Stach-Lempinen B, Eriksson JG (2015). Nutrient intake of pregnant women at high risk of gestational diabetes. Food Nutr Res.

[11] Bowers K, Tobias DK, Yeung E, Hu FB, Zhang C (2012). A prospective study of prepregnancy dietary fat intake and risk of gestational diabetes. Am J Clin Nutr.

[12] Zhang C, Liu S, Solomon CG, Hu FB (2006). Dietary fiber intake, dietary glycemic load, and the risk for gestational diabetes mellitus. Diabetes Care.

[13] Looman M, Schoenaker DAJM, Soedamah-Muthu SS, Geelen A, Feskens EJM, Mishra GD (2018). Pre-pregnancy dietary carbohydrate quantity and quality, and risk of developing gestational diabetes: the Australian Longitudinal Study on Women's Health. Br J Nutr.

[14] Radesky JS, Oken E, Rifas-Shiman SL, Kleinman KP, Rich-Edwards JW, Gillman MW (2008). Diet during early pregnancy and development of gestational diabetes. Paediatr Perinat Epidemiol.

[15] Asadi M, Shahzeidi M, Nadjarzadeh A, Hashemi Yusefabad H, Mansoori A (2019). The relationship between pre-pregnancy dietary patterns adherence and risk of gestational diabetes mellitus in Iran: a case-control study. Nutr Diet.

[16] Du HY, Jiang HOK, Chen B, Xu LJ, Liu SP, Yi JP, He GS, Qian X (2017). Association of dietary pattern during pregnancy and gestational diabetes mellitus: a prospective cohort study in Northern China. Biomed Environ Sci.

[17] Sedaghat F, Akhoondan M, Ehteshami M, Aghamohammadi V, Ghanei N, Mirmiran P, Rashidkhani B (2017). Maternal dietary patterns and gestational diabetes risk: a case-control study. J Diabetes Res.

[18] Schoenaker DAJM, Soedamah-Muthu SS, Callaway LK, Mishra GD (2015). Pre-pregnancy dietary patterns and risk of gestational diabetes mellitus: results from an Australian population-based prospective cohort study. Diabetologia.

[19] Hu J, Oken E, Aris IM, Lin PD, Ma Y, Ding N, Gao M, Wei X, Wen D (2019). Dietary patterns during pregnancy are associated with the risk of gestational diabetes mellitus: evidence from a Chinese prospective birth cohort study. Nutrients.

[20] Tryggvadottir EA, Medek H, Birgisdottir BE, Geirsson RT, Gunnarsdottir I (2016). Association between healthy maternal dietary pattern and risk for gestational diabetes mellitus. Eur J Clin Nutr.

[21] Mak JKL, Pham NM, Lee AH, Tang L, Pan X, Binns CW, Sun X (2018). Dietary patterns during pregnancy and risk of gestational diabetes: a prospective cohort study in Western China. Nutr J.

[22] Yong HY, Mohd Shariff Z, Mohd Yusof B, Rejali Z, Appannah G, Bindels J, Tee YYS, van der Beek EM (2020). The association between dietary patterns before and in early pregnancy and the risk of gestational diabetes mellitus (GDM): Data from the Malaysian SECOST cohort. PLoS ONE.

[23] Phillips CM, Chen L, Heude B, Bernard JY, Harvey NC, Duijts L, Mensink-Bout SM, Polanska K, Mancano G, Suderman M, Shivappa N, Hébert JR (2019). Dietary inflammatory index and non-communicable disease risk: a narrative review. Nutrients.

[24] Shivappa N, Hébert JR, Akhoundan M, Mirmiran P, Rashidkhani B (2019). Association between inflammatory potential of diet and odds of gestational diabetes mellitus among Iranian women. J Matern Fetal Neonatal Med.

[25] Loy SL, Chan JKY, Wee PH, Colega MT, Cheung YB, Godfrey KM, Kwek K, Saw SM, Chong Y, Natarajan P, Müller-Riemenschneider F, Lek N, Chong MF, Yap F (2017). Maternal circadian eating time and frequency are associated with blood glucose concentrations during pregnancy. J Nutr.

[26] Plows JF, Stanley JL, Baker PN, Reynolds CM, Vickers MH (2018). The pathophysiology of gestational diabetes mellitus. Int J Mol Sci.

[27] Pellonperä O, Mokkala K, Houttu N, Vahlberg T, Koivuniemi E, Tertti K, Rönnemaa T, Laitinen K (2019). Efficacy of fish oil and/or probiotic intervention on the incidence of gestational diabetes mellitus in an at-risk group of overweight and obese women: a randomized, placebo-controlled. Double-Blind Clinical Trial Diabetes Care.

[28] Working Group Established by the Finnish Medical Society Duodecim. The Medical Advisory Board of the Finnish Diabetes Association. The Finnish Gynecological Association (2013). Gestational diabetes: current care guidelines.

[29] National Institute for Health and Welfare, Public Health Promotion Unit. Fineli. Finnish food composition database. Release 20. Helsinki 2019. In: www.fineli.fi. Accessed 3 Mar 2021

[30] Eating together—food recommendations for families with children. Finnish institute for health and welfare.(2019). In: https://www.julkari.fi/bitstream/handle/10024/137770/URN_ISBN_978-952-343-264-2.pdf?sequence=1&isAllowed=y. Accessed 3 Mar 2021

[31] Leppälä J, Lagström H, Kaljonen A, Laitinen K (2010). Construction and evaluation of a self-contained index for assessment of diet quality. Scand J Public Health.

[32] Shivappa N, Steck SE, Hurley TG, Hussey JR, Hébert JR (2014). Designing and developing a literature-derived, population-based dietary inflammatory index. Public Health Nutr.

[33] Shivappa N, Steck SE, Hurley TG, Hussey JR, Ma Y, Ockene IS, Tabung F, Hébert JR (2014). A population-based dietary inflammatory index predicts levels of C-reactive protein in the Seasonal Variation of Blood Cholesterol Study (SEASONS). Public Health Nutr.

[34] Sen S, Rifas-Shiman SL, Shivappa N, Wirth MD, Hébert JR, Gold DR, Gillman MW, Oken E (2016). Dietary inflammatory potential during pregnancy is associated with lower fetal growth and breastfeeding failure: results from project viva. J Nutr.

[35] Wirth MD, Shivappa N, Khan S, Vyas S, Beresford L, Sofge J, Hébert JR (2020). Impact of a 3-month anti-inflammatory dietary intervention focusing on watermelon on body habitus, inflammation, and metabolic markers: a pilot study. Nutr Metab Insights.

[36] Byrd DA, Judd SE, Flanders WD, Hartman TJ, Fedirko V, Bostick RM (2019). Development and validation of novel dietary and lifestyle inflammation scores. J Nutr.

[37] Harmon BE, Wirth MD, Boushey CJ, Wilkens LR, Draluck E, Shivappa N, Steck SE, Hofseth L, Haiman CA, Le Marchand L, Hébert JR (2017). The dietary inflammatory index is associated with colorectal cancer risk in the multiethnic cohort. J Nutr.

[38] Hébert JR, Shivappa N, Wirth MD, Hussey JR, Hurley TG (2019). Perspective: the dietary inflammatory index (DII)-lessons learned, improvements made, and future directions. Adv Nutr.

[39] Mansikkaniemi K, Juonala M, Taimela S, Hirvensalo M, Telama R, Huupponen R, Saarikoski L, Hurme M, Mallat Z, Benessiano J, Jula A, Taittonen L, Marniemi J, Kähönen M, Lehtimäki T, Rönnemaa T, Viikari J, Raitakari OT (2012). Cross-sectional associations between physical activity and selected coronary heart disease risk factors in young adults. The cardiovascular risk in young finns study. Ann Med.

[40] Pahkala K, Heinonen OJ, Simell O, Viikari JSA, Rönnemaa T, Niinikoski H, Raitakari OT (2011). Association of physical activity with vascular endothelial function and intima-media thickness. Circulation.

[41] Koska J, Ozias MK, Deer J, Kurtz J, Salbe AD, Harman SM, Reaven PD (2016). A human model of dietary saturated fatty acid induced insulin resistance. Metabolism.

[42] Minihane AM, Vinoy S, Russell WR, Baka A, Roche HM, Tuohy KM, Teeling JL, Blaak EE, Fenech M, Vauzour D, McArdle HJ, Kremer BHA, Sterkman L, Vafeiadou K, Benedetti MM, Williams CM, Calder PC (2015). Low-grade inflammation, diet composition and health: current research evidence and its translation. Br J Nutr.

[43] Zhang Z, Wu Y, Zhong C, Zhou X, Liu C, Li Q, Chen R, Gao Q, Li X, Zhang H, Zhang Y, Cui W, Hao L, Wei S, Yang X, Yang N (2021). The association between dietary inflammatory index and gestational diabetes mellitus risk in a prospective birth cohort study. Nutrition.

[44] Mazidi M, Rezaie P, Banach M (2018). Effect of magnesium supplements on serum C-reactive protein: a systematic review and meta-analysis. Arch Med Sci.

[45] Jafarnejad S, Boccardi V, Hosseini B, Taghizadeh M, Hamedifard Z (2018). A meta-analysis of randomized control trials: the impact of vitamin C supplementation on serum CRP and serum hs-CRP concentrations. Curr Pharm Des.

[46] Fatahi S, Pezeshki M, Mousavi SM, Teymouri A, Rahmani J, Kord Varkaneh H, Ghaedi E (2019). Effects of folic acid supplementation on C-reactive protein: A systematic review and meta-analysis of randomized controlled trials. Nutr Metab Cardiovasc Dis.

[47] Weickert MO, Pfeiffer AFH (2008). Metabolic effects of dietary fiber consumption and prevention of diabetes. J Nutr.

[48] Han S, Cheng D, Liu N, Kuang H (2018). The relationship between diabetic risk factors, diabetic complications and salt intake. J Diabetes Complicat.

[49] Ley SH, Hanley AJ, Retnakaran R, Sermer M, Zinman B, O'Connor DL (2011). Effect of macronutrient intake during the second trimester on glucose metabolism later in pregnancy. Am J Clin Nutr.

[50] Bao W, Bowers K, Tobias DK, Hu FB, Zhang C (2013). Prepregnancy dietary protein intake, major dietary protein sources, and the risk of gestational diabetes mellitus: a prospective cohort study. Diabetes Care.

[51] Liang Y, Gong Y, Zhang X, Yang D, Zhao D, Quan L, Zhou R, Bao W, Cheng G (2018). Dietary protein intake, meat consumption, and dairy consumption in the year preceding pregnancy and during pregnancy and their associations with the risk of gestational diabetes mellitus: a prospective cohort study in southwest China. Front Endocrinol (Lausanne).

[52] Zhao L, Wang M, Li J, Bi Y, Li M, Yang J (2019). Association of circulating branched-chain amino acids with gestational diabetes mellitus: a meta-analysis. Int J Endocrinol Metab.

[53] Flanagan A, Bechtold DA, Pot GK, Johnston JD (2021). Chrono-nutrition: From molecular and neuronal mechanisms to human epidemiology and timed feeding patterns. J Neurochem.

[54] Johansson L, Solvoll K, Bjørneboe GE, Drevon CA (1998). Under- and overreporting of energy intake related to weight status and lifestyle in a nationwide sample. Am J Clin Nutr.

[55] Anglé S, Engblom J, Eriksson T, Kautiainen S, Saha M, Lindfors P, Lehtinen M, Rimpelä A (2009). Three factor eating questionnaire-R18 as a measure of cognitive restraint, uncontrolled eating and emotional eating in a sample of young Finnish females. Int J Behav Nutr Phys Act.

[56] Zhou X, Chen R, Zhong C, Wu J, Li X, Li Q, Cui W, Yi N, Xiao M, Yin H, Xiong G, Han W, Hao L, Yang X, Yang N (2018). Maternal dietary pattern characterised by high protein and low carbohydrate intake in pregnancy is associated with a higher risk of gestational diabetes mellitus in Chinese women: a prospective cohort study. Br J Nutr.

