# Supplementary material for: A healthy dietary pattern with a low inflammatory potential reduces the risk of gestational diabetes mellitus
Source: Eur J Nutr. 2021 Nov 30;61(3):1477–90. doi: 10.1007/s00394-021-02749-z (PMC8921111; doi:10.1007/s00394-021-02749-z)
Supplement: Supplementary file 1 — Supplementary file1 (DOCX 30 KB) [file 394_2021_2749_MOESM1_ESM.docx]

**Online Resource 1**

A healthy dietary pattern with low inflammatory potential reduces the risk of gestational diabetes mellitus

European Journal of Nutrition

Lotta Pajunen^1^, Liisa Korkalo, Ella Koivuniemi, Noora Houttu, Outi Pellonperä, Kati Mokkala, Nitin Shivappa, James R. Hébert, Tero Vahlberg, Kristiina Tertti, Kirsi Laitinen

^1^Institute of Biomedicine, Research Centre for Integrative Physiology and Pharmacology, University of Turku, 20520 Turku, Finland

Email: loevpa@utu.fi

FOPP-study

n=439 women participated in their early pregnancies

Did not meet inclusion criteria because of familial hypercholesterolemia

n=1

Filled in three-day food diary in early pregnancy

n=422

Available result from early or mid-pregnancy OGTT

n=385

Excluded: positive test result from early pregnancy OGTT

n=34

Women included in the analysis

n=351

**Fig. 1** Flow chart of the present study

OGTT; oral glucose tolerance test
