# Supplementary material for: A healthy dietary pattern with a low inflammatory potential reduces the risk of gestational diabetes mellitus
Source: Eur J Nutr. 2021 Nov 30;61(3):1477–90. doi: 10.1007/s00394-021-02749-z (PMC8921111; doi:10.1007/s00394-021-02749-z)
Supplement: Supplementary file 2 — Supplementary file2 (DOCX 17 KB) [file 394_2021_2749_MOESM2_ESM.docx]

**Online Resource 2**

A healthy dietary pattern with low inflammatory potential reduces the risk of gestational diabetes mellitus

European Journal of Nutrition

Lotta Pajunen^1^, Liisa Korkalo, Ella Koivuniemi, Noora Houttu, Outi Pellonperä, Kati Mokkala, Nitin Shivappa, James R. Hébert, Tero Vahlberg, Kristiina Tertti, Kirsi Laitinen

^1^Institute of Biomedicine, Research Centre for Integrative Physiology and Pharmacology, University of Turku, 20520 Turku, Finland

Email: loevpa@utu.fi

List of the food groups obtained from three-day food diaries. Bolded 24 food groups were utilized in the dietary pattern analysis.

**Vegetables**

**Potatoes**

**Fruits and berries**

**Rye bread**

**Multigrain and wheat bread**

**Porridge, muesli, cereal**

**Pasta and rice**

**Sweet pastries**

**Fast food**

**Margarine and oils**

**Butter and other fats**

**Eggs**

**Fish and sea food**

**Poultry**

**Meat**

**Dairy (e.g. milk, sour milk, yogurt, quark)**

**Snacks (e.g. nuts, seeds, popcorn, potato crisps, dried fruits)**

**Coffee and tea**

**Beverages**

**Fruit and berry desserts (e.g. jams, purees)**

**Dairy desserts (e.g. ice-cream, whipped cream)**

**Sugar, sweets, chocolate**

**Savory pastries**

**Cheeses**

Substitutes for meat and dairy

Mayonnaise salads

Spices

Alcohol beverages

Beverages with no energy (e.g. coffee and tea without sugar or milk, water)
