# Supplementary material for: A healthy dietary pattern with a low inflammatory potential reduces the risk of gestational diabetes mellitus
Source: Eur J Nutr. 2021 Nov 30;61(3):1477–90. doi: 10.1007/s00394-021-02749-z (PMC8921111; doi:10.1007/s00394-021-02749-z)
Supplement: Supplementary file 3 — Supplementary file3 (DOCX 21 KB) [file 394_2021_2749_MOESM3_ESM.docx]

**Online Resource 3**

A healthy dietary pattern with a low inflammatory potential reduces the risk of gestational diabetes mellitus

European Journal of Nutrition

Lotta Pajunen^1^, Liisa Korkalo, Ella Koivuniemi, Noora Houttu, Outi Pellonperä, Kati Mokkala, Nitin Shivappa, James R. Hébert, Tero Vahlberg, Kristiina Tertti, Kirsi Laitinen

^1^Institute of Biomedicine, Research Centre for Integrative Physiology and Pharmacology, University of Turku, 20520 Turku, Finland

Email: loevpa@utu.fi

Table 1 Clinical characteristics between the women included in the study and the women excluded from the study.

| Characteristics | Included in the study | Not included in the study^e^ | p^a^ |
| --- | --- | --- | --- |
|  | n=351 | n=87 |  |
| Age (years)^b^ | 30.6±4.50 | 30.8 ±4.93 | 0.701 |
| Primipara^c^ | 174 (49.7) | 37 (45.1) | 0.594 |
| University or college education^c^ | 213 (63.0) | 26 (49.1) | 0.068 |
| Blood pressure (mmHg) at early pregnancy study visit:  Systolic^b^  Diastolic^b^ | 117±10  76±8.3 | 117±11  77±8.4 | 0.975  0.367 |
| Pre-pregnancy BMI (kg/m^2^)^d^ | 28.4 (26.4–31.4) | 29.8 (27.1–33.5) | 0.010 |
| Overweight^c^ | 219 (62.4) | 47 (54.0) | 0.177 |
| Obese^c^ | 132 (37.6) | 40 (46.0) |  |
| Smoking before pregnancy^c^ | 71 (20.9) | 15 (28.3) | 0.217 |
| Gestational weeks at early pregnancy study visit^b^ | 13.9±2.1 | 13.5±2.3 | 0.067 |
| Previous GDM^c^ | 20 (5.7) | 20 (23.0) | <0.001 |

^a^ Independent samples t-test for normally distributed variables, Mann-Whitney U test for non-normally distributed variables and Chi-Squared test or Fisher’s exact test for categorical variables

Data are shown as ^b^ mean ± SD, ^c^ frequencies (percentages), ^d^ median (IQR)

^e^ Not fulfilling the inclusion criteria n=87

^*^Significant value (p<0.05)

BMI, body mass index

Overweight BMI<30, Obese BMI≥30
