# Supplementary material for: A healthy dietary pattern with a low inflammatory potential reduces the risk of gestational diabetes mellitus
Source: Eur J Nutr. 2021 Nov 30;61(3):1477–90. doi: 10.1007/s00394-021-02749-z (PMC8921111; doi:10.1007/s00394-021-02749-z)
Supplement: Supplementary file 4 — Supplementary file4 (DOCX 19 KB) [file 394_2021_2749_MOESM4_ESM.docx]

**Online Resource 4**

A healthy dietary pattern with a low inflammatory potential reduces the risk of gestational diabetes mellitus

European Journal of Nutrition

Lotta Pajunen^1^, Liisa Korkalo, Ella Koivuniemi, Noora Houttu, Outi Pellonperä, Kati Mokkala, Nitin Shivappa, James R. Hébert, Tero Vahlberg, Kristiina Tertti, Kirsi Laitinen

^1^Institute of Biomedicine, Research Centre for Integrative Physiology and Pharmacology, University of Turku, 20520 Turku, Finland

Email: loevpa@utu.fi

Table 2 Associations between the joint effects of energy-adjusted nutrient intakes and the risk of gestational diabetes mellitus

|  | OR^b^ | 95% CI | p^a^ |
| --- | --- | --- | --- |
| Model 1 |  |  |  |
| SFA (E%)^c^  Folate diet (ug/MJ)^d^  Vitamin C diet (mg/MJ)^e^  Phosphorus diet (mg/MJ)^d^  Manganese total (mg/MJ)^f^  Fiber (g/MJ)^e^ | 1.32  0.95  1.00  0.95  0.94  0.80 | (0.83–2.09)  (0.56–1.61)  (0.97–1.03)  (0.87–1.04)  (0.81–1.08)  (0.49–1.32) | 0.236  0.858  0.929  0.255  0.375  0.382 |
| Model 2 |  |  |  |
| SFA (E%)^c^  Folate diet (ug/MJ)^d^  Vitamin C diet (mg/MJ)^e^  Phosphorus diet (mg/MJ)^d^  Manganese diet (mg/MJ)^f^ | 1.35  0.90  1.00  0.96  0.88 | (0.86–2.13)  (0.54–1.50)  (0.97–1.03)  (0.87–1.04)  (0.76–1.02) | 0.193  0.687  0.941  0.313  0.097 |
| Model 3 |  |  |  |
| SFA (E%)^c^  Vitamin C diet (mg/MJ)^e^  Magnesium diet (mg/MJ)^d^  Manganese diet (mg/MJ)^f^ | 1.38  1.00  0.91  0.86 | (0.87–2.19)  (0.97–1.02)  (0.56–1.49)  (0.73–1.02) | 0.167  0.878  0.702  0.078 |
| Model 4 |  |  |  |
| SFA (E%)^c^  Vitamin C diet (mg/MJ)^e^  Magnesium diet (mg/MJ)^d^  Manganese total (mg/MJ)^f^ | 1.38  1.00  0.86  0.90 | (0.87–2.19)  (0.97–1.02)  (0.54–1.37)  (0.78–1.04) | 0.167  0.892  0.527  0.147 |
| Model 5 |  |  |  |
| SFA (E%)^c^  Vitamin C diet (mg/MJ)^e^  Potassium diet (g/MJ)^d^  Fiber (g/MJ)^e^  Manganese diet (mg/MJ)^f^ | 1.33  1.00  0.99  0.85  0.88 | (0.84–2.12)  (0.98–1.03)  (0.95–1.03)  (0.52–1.40)  (0.75–1.04) | 0.228  0.860  0.585  0.524  0.132 |
| Model 6 |  |  |  |
| SFA (E%)^c^  Vitamin C diet (mg/MJ)^e^  Potassium diet (g/MJ)^d^  Fiber (g/MJ)^e^  Manganese total (mg/MJ)^f^ | 1.33  1.00  0.99  0.79  0.92 | (0.84–2.11)  (0.98–1.03)  (0.96–1.03)  (0.49–1.28)  (0.80–1.06) | 0.231  0.851  0.607  0.345  0.224 |

^a^ Multivariable logistic regression models. Nutrients chosen in the models were in the same unit, did not correlate with each other and intakes differed significantly between the women developing and women not developing GDM

^b^ Adjusted for pre-pregnancy BMI and original trial intervention groups; OR for ^c^ five-unit, ^d^ ten-unit, ^e^ one-unit or ^f^ 0.1-unit increase in continuous factors

OR, odds ratio; CI, confidence interval; SFA, saturated fatty acid
